# Two new cyclophoroid species from Hubei, China, with proposal of a new genus (Gastropoda, Caenogastropoda, Diplommatinidae and Alycaeidae)

**DOI:** 10.3897/zookeys.935.51414

**Published:** 2020-05-21

**Authors:** Zhe-Yu Chen, Min Wu

**Affiliations:** 1 College of food science and engineering, Wuhan Polytechnic University, Wuhan 430023, China; 2 School of Life Sciences, Nanjing University, Xianlindadao 163, Nanjing 210023, China

**Keywords:** new genus, new species, *
Pincerna
*, *
Sinoarinia
*

## Abstract

Two new species, *Sinoarinia feii* Chen, **gen. et sp. nov.** and *Pincerna vallis* Chen & Wu, **sp. nov.**, are described from western Hubei, China. The new genus *Sinoarinia* Chen & Wu, **gen. nov.** is established based on the strongly constricted penultimate whorl and the ascending last whorl. The Vietnamese species *Pincerna costulosa* (Bavay & Dautzenberg) is newly reported from Yunnan, China. For comparison, photographs of the shells of closely related species are provided. A key to the known species of *Sinoarinia***gen. nov.** is included.

## Introduction

The malacofauna of western Hubei has usually been studied at some well-known localities along the Yangtze River, for example in Badong and Changyang (Heude 1882, 1885, 1890; Yen 1939). In the region farther south, which encompasses vast areas of limestone mountains, the land snail fauna is still poorly known. During a short faunal survey in Wufeng (Tujiazu Autonomous County) in summer 2019, we found two cyclophoroid species new to science, namely *Sinoarinia feii* Chen gen. et sp. nov. (Diplommatinidae) and *Pincerna vallis* Chen & Wu, sp. nov. (Alycaeidae).

*Arinia* H. Adams & A. Adams, 1856 (Diplommatinidae) is a speciose genus widely distributed in Southeast Asia (Zilch 1953; Vermeulen 1996; Vermeulen et al. 2007; Páll-Gergely and Hunyadi 2018). Only three species of this genus were reported from China so far, namely *Arinia cathaicola* Pilsbry, 1934, *Arinia mirifica* Li, Zhuo & Luo, 2005, and *Arinia maolanensis* Zhang, Chen & Zhuo, 2013 (Pilsbry 1934; Li et al. 2005; Zhang et al. 2013). Based on our morphological studies of shells, we believed that *A. mirifica* and *A. maolanensis*, *Diplommatina aesopus* Bavay & Dautzenberg, 1904 from Vietnam, and *S. feii* sp. nov. belong to a distinct, undescribed genus.

## Methods

Photographs of the shells and the habitats were taken using a Canon 5D Mark IV camera. The shells were measured with digital vernier calipers to the nearest 0.1 mm. Whorls were counted as described by Kerney and Cameron (1979). The terminology to describe alycaeid shells (Regions 1–3) follows Páll-Gergely et al. (2017).

Abbreviations: **a.s.l.** – above sea level; **D** – shell breadth; **H** – shell height; **HBUMM** – mollusc collection of the Museum of Hebei University, Baoding, China; **MNHN** – Muséum National d’Histoire Naturelle, Paris, France; **R1** (Region 1) – from the beginning of the teleoconch to the beginning of the differently ribbed region along the suture; **R2** (Region 2) – the differently ribbed area before the constriction; **R3** (Region 3) – from the constriction to the peristome.

## Systematics

### Diplommatinidae Benson, 1849

#### Sinoarinia

Taxon classificationAnimaliaArchitaenioglossaCyclophoridae

Chen & Wu gen. nov.

7114A84B-8708-5039-B69A-CAFA11B9AA6F

http://zoobank.org/97E91F22-0BD2-4A64-A6A9-2811065ED0F4

##### Type species.

*Sinoarinia feii* Chen, sp. nov.

##### Diagnosis.

Shell minute, dextral, thin, translucent, cylindrical. Penultimate whorl strongly constricted. Last whorl strongly ascending, covering part of penultimate whorl. Protoconch oblique. Apical whorls depressed, with strong or weak ribs. Aperture rounded; peristome double, expanded but not reflected. Columellar lamella not visible from aperture but internally present; one parietal lamella and one palatal plica extending inwards from last whorl to penultimate whorl (in type species). Palatal plica visible through semitransparent parietal wall. Constriction absent or very weak where lamellae and plica terminate. Umbilicus closed, forming a chink.

##### Etymology.

Combination of the Latin prefix sino- (= China) with *Arinia*.

##### Distribution.

Southern China (Hubei, Guizhou), northern Vietnam.

##### Remarks.

The new genus can be distinguished from the sympatric diplommatinid genera (*Arinia*, *Diplommatina* Benson, 1849 and *Gastroptychia* Kobelt & Möllendorff, 1900) by its constricted penultimate whorl and the ascending last whorl (Kobelt 1902; Zilch 1953; Vermeulen 1996; Vermeulen et al. 2007; Páll-Gergely and Hunyadi 2018). In general, *Sinoarinia* gen. nov. has a cylindrical shell, depressed apex, and deeply incised lamellae and plica, which differs from the conical shell with an exposed columellar lamella in *Diplommatina* and *Gastroptychia*. *Arinia* usually lacks internal apertural barriers (Kobelt 1902; Vermeulen 1996; Vermeulen et al. 2007), which are well developed in *Sinoarinia* gen. nov. The constricted structure of the new genus is similar to that found in the non-sympatric diplommatinid genus *Diancta* E. von Martens, 1864, which differs from the new genus by its mostly sinistral coiling and conical whorls as showed in *Diancta* (s. str.) (Neubert and Bouchet 2015), or by the constricted penultimate whorl which is not partially covered by the last whorl, as showed in Diancta (Paradiancta) Möllendorff, 1895 (Egorov 2013).

Three species, *Arinia mirifica* Li, Zhuo & Luo, 2005, *A. maolanensis* Zhang, Chen & Zhuo, 2013, and *Diplommatina aesopus* Bavay & Dautzenberg, 1904, which some authors currently assign to *Arinia* (Li et al. 2005; Zhang et al. 2013) and *Diplommatina* (Bavay & Dautzenberg, 1904), are here transferred to *Sinoarinia* gen. nov.

##### Included species.

*Sinoarinia aesopus* (Bavay & Dautzenberg, 1904) comb. nov., *Sinoarinia feii* Chen sp. nov., *Sinoarinia maolanensis* (Zhang et al., 2013) comb. nov., and *Sinoarinia mirifica* (Li et al., 2005) comb. nov.

##### Vernacular name.

华阿勇螺属

#### Sinoarinia feii

Taxon classificationAnimaliaArchitaenioglossaCyclophoridae

Chen, gen. et sp. nov.

C53955C1-FD88-58FF-AA77-CB7BB7D8B782

http://zoobank.org/CD2C6088-75C0-430D-92CD-5BEB49BD06E8

**Figure 1.**
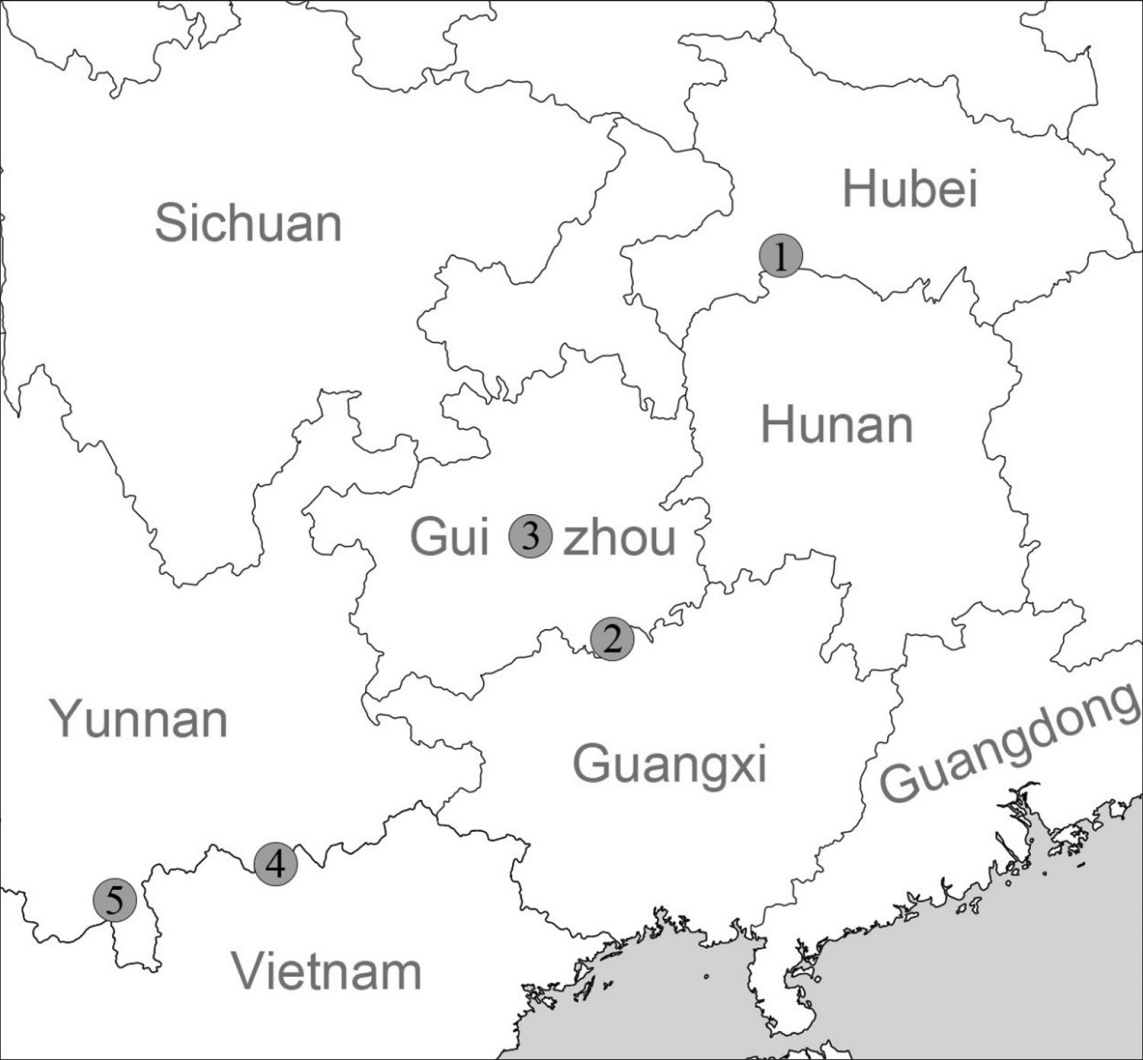
Map of sampling sites and type localities **1** type locality of *Sinoarinia feii* Chen, gen. et sp. nov. and *Pincerna vallis* Chen & Wu, sp. nov. **2** type locality of *Sinoarinia maolanensis* (Zhang, Chen & Zhuo, 2013) comb. nov. **3** type locality of *Sinoarinia mirifica* (Li, Zhuo & Luo, 2005) comb. nov. **4** type locality of *Pincerna costulosa* (Bavay & Dautzenberg, 1912) and *Sinoarinia aesopus* (Bavay & Dautzenberg, 1904) comb. nov., Vietnam **5** Sampling site of *Pincerna costulosa* in Yunnan.

**Figure 2.**
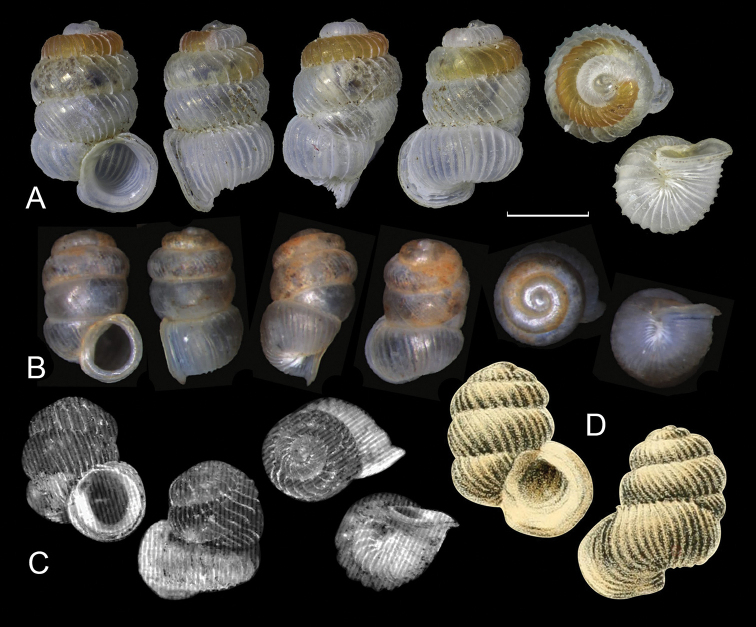
**A***Sinoarinia feii* Chen, gen. et sp. nov. (HBUMM 10016-spec. 1, holotype) (Photos: Zhe-Yu Chen) **B***Sinoarinia maolanensis* (Zhang et al., 2013) comb. nov. (after Zhang et al. 2013) **C***Sinoarinia mirifica* (Li et al., 2005) comb. nov. (after Li et al. 2005) **D***Sinoarinia aesopus* (Bavay & Dautzenberg, 1904) comb. nov. (after Bavay & Dautzenberg, 1904). Scale bar: 1 mm, refers to **A** and **B** only.

**Figure 3.**
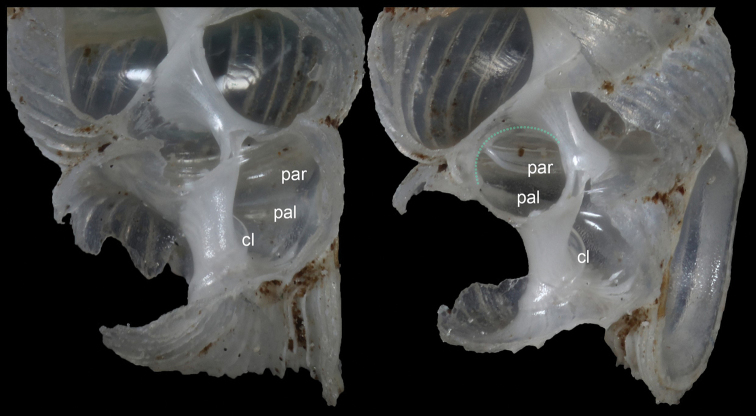
Inner structure of *Sinoarinia feii* Chen, gen. et sp. nov. (HBUMM 10016-spec. 5, paratype) (Photos: Zhe-Yu Chen). Abbreviations: par-parietal lamella, pal-palatal plica, cl-columellar lamella, green dotted line-constriction.

Figures 1 , 2A , 3 , 5B

##### Type material.

***Holotype*** (HBUMM 10016-spec. 1), China, Hubei, Wufeng Tujiazu Autonomous County, Chaibuxi National Forest Park, 30.216N, 110.199E, 1220 m a.s.l., leg. Zhe-Yu Chen and Qiao-Zhen Hu, 27 June 2019 (Fig. 5B). ***Paratypes***: 4 ex. (HBUMM 10016-spec. 2–5), same data as holotype.

##### Measurements.

Shell width = 1.6–1.7 mm, shell height = 2.4–2.7 mm (*n* = 4).

##### Diagnosis.

Shell minute, cylindrical and apically flat. Penultimate whorl strongly constricted. Columellar lamella, one parietal lamella and one palatal plica present.

##### Description.

Shell minute, dextral, cylindrical, translucent, with 5½ whorls. Shell suture depressed. Protoconch oblique, with no obvious sculpture. Upper whorls depressed. Penultimate whorl so strongly constricted that ½ whorl is invisible. Last whorl strongly ascending, dorsally covering part of penultimate whorl. Ribs strong and sharp, concentrated around umbilical region. Aperture rounded. Peristome double, expanded but not reflected. Angular protrusion weakly present near basal columella. Columellar lamella invisible from aperture, but stronger at penultimate whorl. One parietal lamella and one palatal plica extending from last whorl to penultimate whorl (Fig. 3). Palatal plica visible through semi-transparent parietal wall; obvious constriction absent or very weakly bulged at dorsal side of penultimate whorl. Umbilicus closed, chink-shaped. Operculum unknown.

##### Etymology.

This name honors the herpetologist, Prof. Liang Fei, who encouraged the first author to carry out the research work.

##### Type locality.

China, Hubei, Wufeng Tujiazu Autonomous County, Chaibuxi National Forest Park, 30.216N, 110.199E, 1220 m a.s.l.

##### Distribution.

This species is known only from the type locality.

##### Remarks.

*Sinoarinia maolanensis* (Zhang et al. 2013) is morphologically similar, but differs in having a larger shell with stronger ribs (Fig. 2B). *Sinoarinia mirifica* (Li et al. 2005) can be distinguished by its stout shape of shell, sparser ribs, shorter and fewer whorls, and in that the last whorl covers the penultimate whorl dorso-laterally (Fig. 2C) (in *S. feii* gen. et sp. nov. the last whorl covers penultimate whorl dorsally).

##### Vernacular name.

费氏华阿勇螺

### Key to the species of *Sinoarinia* gen. nov.

**Table:** 

1	Shell height greater than 3 mm	***S. aesopus* (Bavay & Dautzenberg, 1904) (Fig. 2D)**
–	Shell height less than 3 mm	**2**
2	Whorls fewer than 5. Shell width / shell height approximately 0.9	***S. mirifica* (Li et al. 2005) (Fig. 2C)**
–	Whorls more than 5. Shell width / shell height approximately 0.5–0.7	**3**
3	Shell height greater than 2 mm. Shell ribs strong	***S. feii* Chen sp. nov. (Fig. 2A)**
–	Shell height less than 2 mm. Shell ribs weak	***S. maolanensis* (Zhang et al. 2013) (Fig. 2B)**

### Alycaeidae Blanford, 1864

#### Pincerna

Taxon classificationAnimaliaArchitaenioglossaCyclophoridae

Preston, 1907

72CDCB32-DD2C-5DDC-BCBB-F1A0E347C0C1

##### Type species.

Alycaeus (Pincerna) liratula Preston, 1907, by monotypy (Páll-Gergely 2017).

##### Vernacular name.

平沟螺属

#### Pincerna vallis

Taxon classificationAnimaliaArchitaenioglossaCyclophoridae

Chen & Wu sp. nov.

1F65093B-37E5-5120-936A-F631EE806136

http://zoobank.org/63A4E519-86E7-453A-9F90-2EAAFE527185

**Figure 4.**
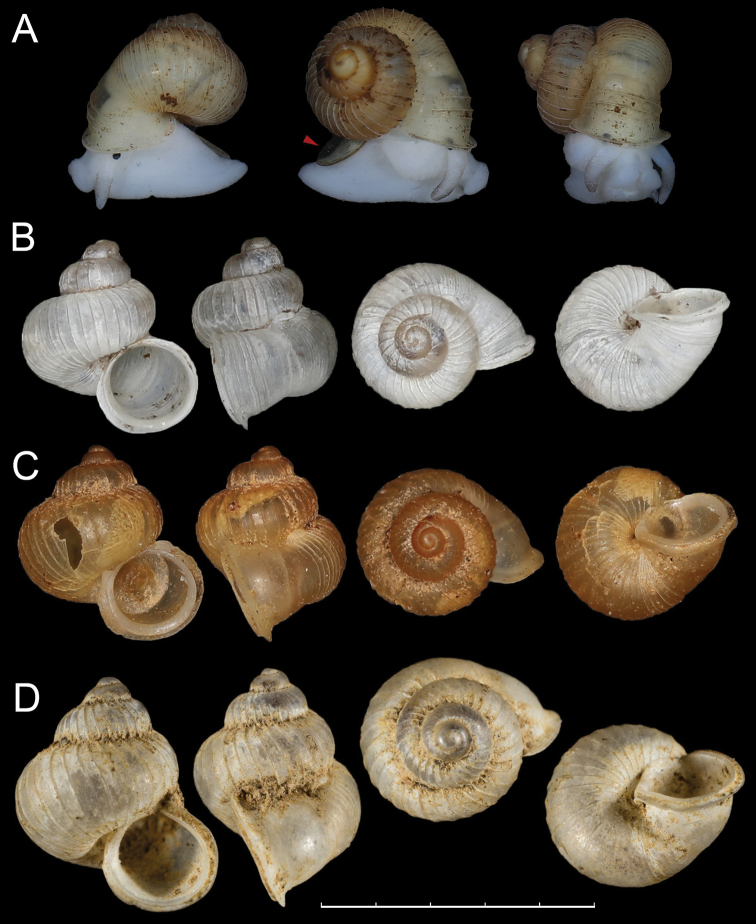
**A, B***Pincerna vallis* Chen & Wu, sp. nov. **A**HBUMM 10017-spec. 1, holotype, red arrow shows the operculum **B**HBUMM 10017-spec. 2, paratype **C, D***Pincerna costulosa* (Bavay & Dautzenber﻿g, 1912) **C** HBUMM10018 **D** syntype, MNHN-IM-2000-31786. Scale bar: 5 mm. Photos: Zhe-Yu Chen (**A–C**) and M. Caballer Gutierrez (**D**).

**Figure 5.**
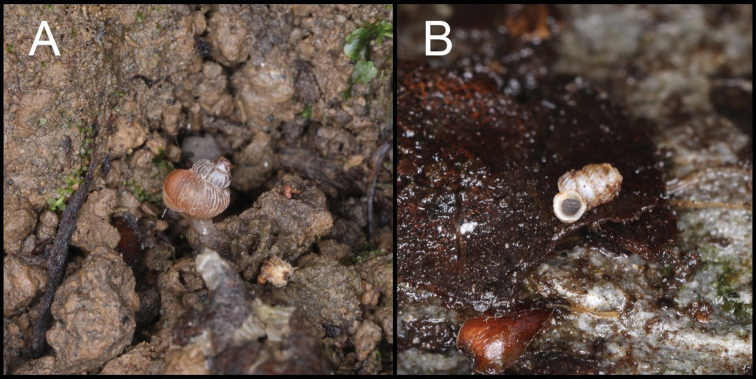
**A** Habitat of *Pincerna vallis* Chen & Wu, sp. nov. (HBUMM 10017-spec. 1, holotype) **B** habitat of *Sinoarinia feii* Chen, gen. et sp. nov. Photos: Zhe-Yu Chen.

Figures 1 , 4A, B , 5A

##### Type material.

***Holotype*** (HBUMM10017-spec. 1, fully mature animal), China, Hubei, Wufeng Tujiazu Autonomous County, Chaibuxi National Forest Park, 30.216N, 110.199E, 1220 m a.s.l., leg. Zhe-Yu Chen and Qiao-Zhen Hu, 27 June 2019 (Fig. 5A). ***Paratype***: 1 ex. (HBUMM 10017-spec. 2, an empty shell), same data as holotype.

**Measurements.** Shell width = 3.3 mm, shell height = 3.5 mm.

**Diagnosis.**R3 with some ribs. Ribs on R2 more intensive than those in *P. costulosa*.

**Description.** Shell conical ovoid, orangish when fresh, with 3¼–3½ convex whorls. Suture deep. Protoconch finely granulate, 1–1¾ whorls. R1 ca 2½ whorls, with regularly spaced strong ribs. R2 very short (ca 0.5 mm), consisting of ca 15 lighter stripes (= breathing tunnels); constriction between R2 and R3 rather shallow. R3 slightly less than ¼ whorl, smooth near R2 side, with about 5 weak but distinct ribs near aperture. Aperture rounded, nearly vertical, not descending. Peristome expanded but not reflected, internally thickened, protruding; boundary between inner and outer peristomes visible. Umbilicus open, narrow. Operculum (Fig. 4A, arrowed) corneous, translucent, thin, concave.

**Etymology.** The name *vallis* (Latin: valley) refers to the type locality inside the Chaibuxi Grand Canyon.

**Type locality.** China, Hubei, Wufeng Tujiazu Autonomous County, Chaibuxi National Forest Park, 30.216N, 110.199E, 1220 m a.s.l.

**Distribution.** This species is known only from the type locality.

**Remarks.** Two *Pincerna* species have been recorded in adjacent areas, *P. costulosa* (Bavay & Dautzenberg, 1912) from Vietnam (Holotype: MNHN-IM-2000-31786, Tonkin, Phong Tho) (Fig. 4D) and Yunnan, China (HBUMM10018, see below) (Fig. 4C) and *Pincerna maolanensis* Luo, Zhang & Zhuo, 2009 from Guizhou. *Pincerna vallis* sp. nov. can be distinguished from *P. maolanensis* by its smaller and more fragile shell, relatively short R2 (ca 0.5 mm, whereas it is 0.83–1.00 mm in *P. maolanensis*). *Pincerna costulosa* is most similar to the new species in size and shape. However, *P. vallis* sp. nov. has a more convex body whorl. *Pincerna costulosa* has a smooth/ribless R3 while the new species has some ribs near the aperture. In addition, the ribs of *P. vallis* sp. nov. on R2 are stronger than those in *P. costulosa*.

##### Vernacular name.

峡谷平沟螺

#### Pincerna costulosa

Taxon classificationAnimaliaArchitaenioglossaCyclophoridae

(Bavay & Dautzenberg, 1912)

675E6B18-3285-5215-9A0A-C73FD5087A2C

Figures 1 , 4C, D

Alycaeus costulosus Bavay & Dautzenberg, 1912: 49–50, pl. 4, figs 1–4.
Pincerna costulosa -Páll-Gergely et al. 2017: 10, fig. 3F.

##### Materials examined.

HBUMM10018 (Fig. 4C), Yunnan, Xishuangbanna, Menglun Botanical Garden, Lvshilin (21.911N, 101.283E), leg. Xiao-Ran Zhu, 6 August 2018.

##### Type locality.

“Phong-Tho, Tonkin”.

##### Distribution in China.

Yunnan.

##### Vernacular name.

弱肋平沟螺

## Supplementary Material

XML Treatment for Sinoarinia

XML Treatment for Sinoarinia feii

XML Treatment for Pincerna

XML Treatment for Pincerna vallis

XML Treatment for Pincerna costulosa

## References

[B1] Bavay A Dautzenberg P (1904 [«1903»]) Description de coquilles nouvelles de l’Indo-Chine (3e suite). Journal de Conchyliologie 51(3): 201–236.

[B2] Bavay A Dautzenberg P (1912) Description de coquilles nouvelles de l’Indo-Chine. Journal de Conchyliologie 60(1): 1–54.

[B3] Egorov R (2013) A review of the genera of the terrestrial pectinibranch molluscs (synopsis mainly based on published data). Littoriniformes: Liareidae, Pupinidae, Diplommatinidae, Alycaeidae, Cochlostomidae. Treasure of Russian Shells, Supplement 3(3): 1–62.

[B4] Heude PM (1882) Notes sur les mollusques terrestres de la vallée du fleuve Bleu. Mémoires Concernant l’Histoire Naturelle de l’Empire Chinois 1: 1–84. 10.5962/bhl.title.50365

[B5] Heude PM (1885) Notes sur les mollusques terrestres de la vallée du fleuve Bleu. Mémoires Concernant l’Histoire Naturelle de l’Empire Chinois 3: 89–132.

[B6] Heude PM (1890) Notes sur les mollusques terrestres de la vallée du fleuve Bleu. Mémoires Concernant l’Histoire Naturelle de l’Empire Chinois 4: 125–188.

[B7] Kerney MP Cameron RAD (1979) A field guide to the land snails of Britain and North-West Europe. Collins, London, 288 pp.

[B8] Kobelt W (1902) Das Tierreich. Eine Zusammenstellung und Kennzeichnung der rezenten Tierformen. Verbindung mit der Deutschen Zoologischen Gesellschaft herausgegeben von der Königlich Preussischen Akademie der Wissenschaften zu Berlin. Mollusca: Cyclophoridae. R. Friedländer und Sohn, Berlin, 662 pp.

[B9] Li Y Zhou W Luo T (2005) A new species of Cyclophoridae from Guizhou Province, China, (Prosobranchia: Mesogastropoda: Cyclophoridae). Acta Zootaxonomica Sinica 30(1): 67–69.

[B10] Luo TC Zhang WH Zhuo WC (2009) A new species of the genus *Dioryx* Benson from China (Prosobranchia, Mesogastropoda, Cyclophoridae). Acta Zootaxonomica Sinica 34(4): 862–864.

[B11] Neubert E Bouchet P (2015) The Diplommatinidae of Fiji–a hotspot of Pacific land snail biodiversity (Caenogastropoda, Cyclophoroidea). ZooKeys 487: 1–85. 10.3897/zookeys.487.8463 PMC4366685 25829849

[B12] Páll-Gergely B (2017) A new species of Alycaeidae, *Pincerna yanseni* sp. nov. from Sumatra, with the resurrection of the genus *Pincerna* Preston, 1907 (Gastropoda: Cyclophoroidea). Raffles Bulletin of Zoology 65: 213–219.

[B13] Páll-Gergely B Hunyadi A (2018) Four new cyclophoroid species from Thailand and Laos (Gastropoda: Caenogastropoda: Alycaeidae, Diplommatinidae, Pupinidae). Zoosystema 40(3): 59–66. 10.5252/zoosystema2018v40a3

[B14] Páll-Gergely B Hunyadi A Đỗ ĐS Naggs F Asami T (2017) Revision of the Alycaeidae of China, Laos and Vietnam (Gastropoda: Cyclophoroidea) I: the genera *Dicharax* and *Metalycaeus*. Zootaxa 4331(1): 1–124. 10.11646/zootaxa.4331.1.1 29242453

[B15] Pilsbry HA (1934) Zoological results of the Dolan West China Expedition of 1931, part II, mollusks. Proceedings of the Academy of Natural Science of Philadelphia 86: 5–28.

[B16] Vermeulen JJ (1996) Notes on the non-marine molluscs of the island of Borneo 8, the genus *Arinia*; additions to the genera *Diplommatina* and *Opisthostoma* (GastropodaProsobranchia: Diplommatinidae). Basteria 60(4–6): 87–138.

[B17] Vermeulen JJ Phung LC Truong QT (2007) New species of terrestrial molluscs (Caenogastropoda, Pupinidae and Pulmonata, Vertiginidae) of the Hon Chong-Ha Tien limestone hills, Southern Vietnam. Basteria 71(1–3): 81–92.

[B18] Yen TC (1939) Die chinesischen Land- und Süßwasser-Gastropoden des Natur-Museums Senckenberg. Abhandlungen der Senckenbergischen Naturforschenden Gesellschaft 444: 1–234. [16 pls.]

[B19] Zhang W Chen D Zhou W (2013) A new species of the genus *Arinia* H. and A. Adams from China (Prosobranchia: Caenogastropoda: Diplommatinidae). Acta Zootaxonomica Sinica 38(4): 773–775.

[B20] Zilch A (1953) Die Typen und Typoide des Natur-Museums Senckenberg, 9: Mollusca, Cyclophoridae, Diplommatininae. Archiv für Molluskenkunde 82(1/3): 1–47.

